# The Treatment of Cancer1President’s address before the fortieth annual meeting of the Medical Association of Central New York, held at Rochester, October 15, 1907.

**Published:** 1908-01

**Authors:** William B. Jones

**Affiliations:** Rochester, N. Y.


### BUFFALO MEDICAL JOURNAL.

VOL. LXIII. JANUARY, 1908. No. 6

### ORIGINAL COMMUNICATIONS.

### The Treatment of Cancer.[1]

BY WILLIAM B, JONES, M. D., Rochester, N. Y.

THE treatment of cancer has attained a measure of success that calls for a new adjustment of the estimate in which it has been held by those of us who knew its limitations twenty years ago. Our members who left college recently will not realise the difference between then and now. Before that period results were so often disappointing that conscientious physicians could only give half-hearted support to some legitimate attempts to remove the disease and none at all to most.

In any case where an operation or extensive or dreaded treatment is suggested without enthusiasm the suggestion is received without much hope. This is why some physicians find their advice so often rejected. Almost every one has confidence and does as told by the doctor who says "You need," certain treatment, "It is the only proper way. I will arrange for it." But there is no such coöperation with the one who says, "I don't know but you will have to do" this or that, "Perhaps it would be better. I'll see tomorrow."

The people generally have faith in the medical profession and in all things where we can demonstrate ability to cure they come to us first,—most of them do,—but when we fail them they are not giving up and being reconciled to abandon all offered help on the strength of our decision that nothing more can be done, and they try all offers, which means rich pickings for quacks. Twenty years ago cancer quacks and institutions were numerous and growing more so. Some of them have gone into a decline and there are few new ones. That is significant that now the people are better satisfied with us than with them.
cession, and if possible regain our proper place. The proper

The men who are only entering practice and those who are

1. President's address before the fortieth annual meeting of the Medical Association of Central New York, held at Rochester, October 15, 1907.

yet building theirs up have perhaps heard enough about cancer for a time, but we who have been here only a little longer are still doing most of the business, are still seeing a good many patients and by your sufferance, young gentlemen, will for a while longer. We need to consider that the glowing oratory of commencements is well founded and that today prepares better than some time ago and it is well if we have kept abreast of modern teachings in the yesterdays between. If we have been at all neglectful it behooves us now to catch up with the procession, and if possible regain our proper place. The proper place for a live man is near the front and he should keep getting nearer.

After all, it makes little difference to the community at large whether a doctor keeps himself keen and up to date or a younger one more ambitious and efficient supplants him, but it does make a tremendous difference whether we are apathetic to what can be done for cancer. The subject is so important that we must take advantage of every slightest resource and we must know and hold the correct estimate of all.

The alarming increase of cancer is no delusion of imaginative statisticians, nor is it only apparent and due to more thorough registration of deaths by health boards, or the present greater dissemination of all kinds of information. Cancer deaths are increasing rapidly in actual numbers, in proportion to other deaths and in proportion to population; also the rate of increase is growing. In this state ten years ago there were each year 50 to 200 more than the year before. Now the increase is 250 to 350 each year.

Of the men present who are 35 or more years of age one in eleven or twelve will die of cancer and of the women one in seven or eight, unless better cared for than our patients have been until the present time. There is better care to give them and you can learn the details of these betterments from the many papers now appearing from authorities who are doing their utmost to inform the profession.

The most important principles upon which the details are worked out are: cancer is prone to develop where there is continuous or frequent irritation. Avoid such an excitant and repair old injuries, such as lacerations, scars where exposed to friction, and the like, benign tumors sometimes become malignant after many years. Remove all neoplasms, especially warts, moles, and fibroid growths.

Cancer is always local at first and curable if all the original growth can be removed before any is transplanted to other places. It is almost always conveyed by lymphatic vessels to nearest lymphatic glands where it is halted for a time, and there is no enlargement or other appearance to indicate this until some time after it occurs, so that removal of the primary growths and all enlarged lymphatics is not enough. All lymphatics leading directly from it, whether enlarged or not, must be removed, and further the chain of glands into which they empty.

The secondary growths, where migrating cancer cells have halted in lymphatic glands and grown, act precisely as the primary ones do, extension from them is in the same manner, and with them must be removed lymphatics leading from them, and the glands into which they empty the same, as in treatment of the primary growths.

That will be the series next deeper, and frequently also another series not deeper but near by with which there is anastomamosis. Operation secures these essentials better than any other treatment and except for very superficial warts and inoperable cases should always be undertaken at the earliest possible moment.

Any tumor, lump or swelling, however small, that has been permitted to remain after it was first noticed must be removed immediately when it begins to grow or cause irritation. It must be carefully examined and if malignant the whole region must be operated on right away for cancer. There must be no waiting to see whether it will return. This replaces the custom of taking a small piece for examination, which should never be done because even the little cutting that requires, opens lymphatics and other vessels and cuts through tumor tissue out of which will exude cells which may enter those vessels, be carried to a distance and, if cancer, implant metastases.

Recurrence after operation should be operated for immediately when it appears according to the same principles as the primary operation and just as thoroughly, and this repeated with every recurrence if in an accessible part. There will be some successes by so doing that would have been failures if we had stopped trying after the first operation. By ligating bloodvessels supplying a cancer, or cancer region growth can be retarded, recurrence delayed and assistance given to other measures, such as cautery, for cure. The growth should not be watched at all for any reason before operation. It should be removed as soon as found and watched afterwards.

Cancer of the viscera is now recognised only by the symptoms of the late and hopeless stages, coffee ground vomit, acidity, fermentation, dilation and pain in the stomach; tumor, jaundice and pain about the gall bladder; tumor, pain and functional disturbance in the intestines; pain and chronic obstruction at the rectum with bloody discharges. These symptoms and cachexia do not occur until cancer has passed through the greater part of its course, indeed, part of them denote septicemia from absorption at places where sloughing has been going on some time. But cancer in these organs causes earlier symptoms that if recognised when they first appear would permit cure of a large proportion of these patients who now are almost all left without a diagnosis until too late. Their salvation depends upon earlier diagnosis and that upon attention to what we too often miscall functional disorder. That patient may have a functional disorder but too often the symptoms are due to organic disease, cancer or something else, and the conservative thing is to consider it organic and to find out what kind of organic disease it is if two weeks of proper and satisfactory treatment results in moderate improvement only, or less.

Many internal organic diseases can only be diagnosticated by exploratory incision, and most of them can only be cured by operation.

525 Lake Aveune.

